# Risk factors for delayed isolation of tuberculosis patients in a tertiary care hospital in a low-incidence country

**DOI:** 10.1186/2047-2994-4-S1-P93

**Published:** 2015-06-16

**Authors:** J Severin, S Ruhe-van der Werff, M Bakker, MC Vos

**Affiliations:** 1 Erasmus MC, Rotterdam, Netherlands

## Introduction

Delayed isolation of tuberculosis patients increases the risk of nosocomial transmission.

## Objectives

The purpose of this study was to analyze risk factors for delayed isolation of tuberculosis patients in a tertiary care hospital in the Netherlands.

## Methods

We included all patients with culture-proven *Mycobacterium tuberculosis* infection between January 1^st^ 2010 and December 31^st^ 2012. Demographic and clinical information was collected retrospectively from the medical records. Risk factors were analyzed using univariate and multivariate logistic regression. A prediction model for non-isolation was developed.

## Results

In the three-year time period, 58 patients had a culture-proven tuberculosis infection in our hospital, 20 with an extrapulmonary infection only. For 41 patients (70.7%), isolation measures were not taken. Twelve of these were patients with extrapulmonary tuberculosis only, and in these cases isolation measures were forgotten with aerosol generating procedures. The median number of days of non-isolation was 4 (range 1-162). For the patients with pulmonary tuberculosis (38 patients, 25 (65.8%) not isolated) four factors were predictive for non-isolation: presence of the symptom “fatigue”, absence of hemoptysis, absence of tuberculosis in the differential diagnosis, absence of involvement of a pulmonologist or infectious diseases specialist on admission. Due to these non-isolated patients, 1115 heath care workers and 78 patients had to be screened for tuberculosis.

## Conclusion

In a low-incidence setting, it seems that tuberculosis patients with a clinical presentation that is not “classical”, tuberculosis is not included in the differential diagnosis, and experts in tuberculosis are not consulted. This leads to delayed isolation of patients, which is a risk for health care workers and other patients.

## Disclosure of interest

None declared.

